# Towards social acceptability of genome-edited plants in industrialised countries? Emerging evidence from Europe, United States, Canada, Australia, New Zealand, and Japan

**DOI:** 10.3389/fgeed.2022.899331

**Published:** 2022-08-31

**Authors:** Armin Spök, Thorben Sprink, Andrew C. Allan, Tomiko Yamaguchi, Christian Dayé

**Affiliations:** ^1^ Science, Technology and Society Unit, Graz University of Technology, Graz, Austria; ^2^ Institute for Biosafety in Plant Biotechnology, Julius Kühn-Institut, Federal Research Center for Cultivated Plants, Quedlinburg, Germany; ^3^ New Cultivar Innovation, Plant & Food Research, Auckland, New Zealand; ^4^ School of Biological Sciences, University of Auckland, Auckland, New Zealand; ^5^ College of Liberal Arts, International Christian University, Tokyo, Japan

**Keywords:** gene-editing, genome editing, acceptability, public perception, stakeholder, policy development, GMO legislation, GMO policy

## Abstract

The agricultural biotechnology world has been divided into two blocks; countries adopting GM crops for commercial cultivation (adopters) and others without any or without relevant cultivation of such crops (non-adopters). Meanwhile, an increasing number of adopter countries have exempted certain genome-edited (GE) crops from legal GMO pre-market approval and labelling requirements. Among them are major exporters of agricultural commodities such as United States, Canada, and Australia. Due to the relaxed legislation more GE plants are expected to enter the market soon. Many countries in the non-adopter group, however, depend on import of large volumes of agricultural commodities from adopter countries. Unlike first generation GM, certain GE crops cannot be identified as unambiguously originating from genome editing using available techniques. Consequently, pressure is mounting on non-adopter jurisdictions to reconsider their policies and legislations. Against this backdrop, the paper explores recent developments relevant for social acceptability in selected non-adopters, Japan, New Zealand, the EU, Norway, and Switzerland in contrast to United States, Canada, and Australia. While Japan is already opening-up and Norway and Switzerland are discussing revisions of their policies, the EU and New Zealand are struggling with challenges resulting from high court decisions. In an attempt to take a closer look into the inner dynamics of these developments, the concept of social acceptability proposed by Wüstenhagen et al. (Energy Policy, 2007, 35(5), 2683–2691) is employed. This aids the understanding of developments in the jurisdictions considered and identifies specific or cross-cutting challenges.

## Introduction

For more than 20 years the overall legal environment for, as well as stakeholder and public views on, genetically modified organisms (GMOs) have been relatively stable: countries in North- and South America, Australia, and certain parts of Asia have developed more enabling regulatory regimes, and in these regions, GM crops have rapidly captured significant market shares. Between 1996 and 2019 global area of GM crops increased from 1.7 to 190.4 million hectares (ISAAA, 2019). According to an estimate by Nature in 2013 this corresponds to a 13% share of cultivated arable land (Nature, 2013). Japan, New Zealand, Norway, Switzerland and the EU, established more restrictive regimes and thus, cultivation and commercial use in food of GM crops have been either slow or severely inhibited. These different regulatory regimes are also reflected by the numbers of GM plant events for which market approvals for cultivation has been granted (see Table 2). In particular, in the EU where publics show a more negative attitude as compared to, for instance, the United States (European Commission, 2006; European Commission, 2010), approval numbers for cultivation are very low. Even if GM plants were approved, this does not necessary imply social and market acceptance by the food value chain actors and/or consumers. A striking example for this is Japan, where essentially no commercial cultivation occurs despite the number of events authorised which is similar to Canada. For the purpose of this review it is pertinent to distinguish these two groups based on their socio-legal acceptance of GM plants for cultivation. They will be referred to as adopters and non-adopters.

The differences in the socio-political environments also affected approvals for GM food and feed—though to less extent (see Table 3). Some jurisdictions invoked a zero-tolerance policy for non-authorised events, other allowed for trace amounts. Consequently, international trade of food and feed commodities has turned out to be complex. Despite this challenging environment for global trade, a kind of working routine emerged for farmers, importers and food/feed producers guided by coexistence rules, food control and separation of supply chains.

In recent years, the advent of genome-edited organisms (GEOs) is posing new challenges to these arrangements: an increasing number of GMO-adopters such as the United States, Canada, Brazil, Argentina, and Australia have exempted genome-edited plants of SDN1-type (small insertions or deletions which carry no additional or recombinant DNA) and derived food and feed from their GMO legislation or allowed commercialisation based on a simplified case-by-case procedure (Eckerstorfer et al., 2019; Eriksson et al., 2019; Menz et al., 2020; Entine et al., 2021; Turnbull et al., 2021). This has sparked the development of new plant varieties, and a range of genome-edited plant products with minor genetic changes are expected to enter global commodity markets soon (reviewed in Menz et al., 2020; Parisi and Rodríguez-Cerezo 2021). As certain types of genome-edited products cannot be analytically identified as originating from genome editing (Grohmann et al., 2019), food control in jurisdictions where genome-edited plants require pre-market authorisation and labelling cannot guarantee that the existing legislation can be enforced in the future. Such a scenario is likely to be associated with considerable uncertainties and business risks for the food and feed value chains. In particular food importers, food producers, and retailers might be confronted with reports or criticisms from GMO-critical groups that certain ingredients in their products are genome-edited and illegal on the market. As a consequence, this could lead to recalls of products, negative press, diminished consumer trust and potential liability issues, disrupting agricultural as well as food and feed supply chains. Although still a hypothetical scenario—pressure is mounting on jurisdictions that treat these GE products in the same way as GMOs, including pre-market approval and labelling requirements.

As regards non-adopters, Ishii and Araki (2017) anticipated that this group would split into two, with one developing policies for GEOs along the lines in the United States or Argentina and a second one where the regulations effectively prevent a cultivation of GEO crops, such as New Zealand. The EU, Ishii and Araki hypothesized, would proceed the same way as New Zealand, Japan and South Korea—both with little or no previous adoption in terms of commercial cultivation of GM crops—were predicted to follow the examples in North- or South-America. The United Kingdom also appears to have joined this group with the new legislation (Practical Law Environment, 2022) that is no-longer bound to EU legislations and policies post-Brexit.

**FIGURE 1:**
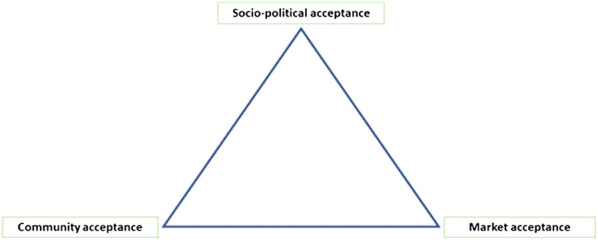
The triangle of social acceptance. Source: Wüstenhagen et al. (2007).

The key factor in the non-adopter group how to proceed in terms of GEO will be social acceptability. Social acceptance is a multi-dimensional phenomenon, comprising legal, social, cultural, historical, and economic aspects. The characteristics and potential benefit of a technology is only one aspect amongst many, and the trajectories of a technology can differ fundamentally in different regions of the world. Acknowledging this multi-dimensionality, Wüstenhagen et al. (2007) proposed a triangle of social acceptance that highlights three dimensions: 1) socio-political acceptance; 2) community acceptance; and 3) market acceptance (see Figure 1). The three dimensions differ in terms of both, subjects and objects of acceptance as detailed in Table 1 (see also Sonnberger and Ruddat, 2017).

**TABLE 1:** Dimensions of social acceptance based on Wüstenhagen et al. (2007) and Sonnberger and Ruddat (2017), their characteristics and what type of evidence is considered relevant and used in this review.

Dimensions	Objects	Subjects	Source of evidence
Socio-political acceptance	Technology and/or its legal regulation	Stakeholders, politicians, and general publics	Public opinion polls, comparative reviews of regulation, observed actions and initiatives by subjects of acceptance
Market acceptance	Specific product or service	Value chain actors, including corporate businesses, investors, and consumers	Consumer studies (willingness-to-pay, willingness-to-consume etc.), market observations
Community acceptance	Specific local project using the technological innovation	Local population, stakeholders concerned by the specific project, and the local administration	Since the focus of the review is on non-adopters, and social scientific studies of concrete projects with GMOs/GEOs in these countries are virtually non-existent, this dimension cannot be covered.

Particularly by making clear that acceptance may concern different things—the technology, its regulation, a specific project, a product or service applying the technology—the triangle of social acceptance allows to capture crucial intricacies that often haunt debates on the social acceptance and cause various misunderstanding. Still, the notion of acceptance has attracted some criticism in the recent decades, most of it not directly related to the points raise by Wüstenhagen et al. (2007). It has been argued that the discussion about social acceptance assumes technological innovation is separated out from the dynamics of society, thus it has been conceptualized that society is to accept (or reject) what has been delivered without having any influences on technological innovation. Meanwhile, it has been widely recognised that this concept falls short of explaining real world challenges of technological innovation. This is reflected, for instance, by the EU’s promotion of the RRI (Responsible Research and Innovation) as a guiding principle and policy of the region. The political connotation of the term acceptance has also been used to indicate different styles in the regulation of GM/GE food (e.g., Meyer, 2017; Meyer, 2020; Meyer and Vergnaud 2021). While this is certainly true for the general use of this term, the disentanglement of the various dimensions of acceptance suggested by Wüstenhagen et al. (2007) precludes such interpretation and increases the descriptive-analytic value of the term.

Further, it has been argued that the term acceptance does not help in understanding the underlying processes by which social acceptance occurs (Szarka, 2007; Fournis and Fortin, 2017; Alexandre et al., 2018). To capture the dynamics and conditions under which a certain technology becomes accepted, a more sustained focus on the processes is required, a point that also had been repeatedly emphasised with regard to plant biotechnologies (Yamaguchi and Harris, 2004; Levidow et al., 2007). Thus, the term *acceptability* captures social dynamics more so than the term *acceptance*.

In light of these considerations, this review adds to the extant literature by relating various strands of research on and debates about the social acceptance of genome-edited plants with the objective to provide a more comprehensive picture of the current dynamics in various jurisdictions. The extant literature is concerned mostly with either legal aspects (e.g., Erikson et al., 2018; Entine et al., 2021; Turnbull et al., 2021) or consumer/citizen perspectives (e.g., Runge et al., 2017; Scott et al.*,* 2018; Siegrist and Hartmann, 2020; Beghin and Gustafson, 2021; Grant et al., 2021). Such perspectives certainly capture the temporal dynamics within a specific dimension of social acceptability (or a small part of a dimension), but not between the dimensions. The paper aims to achieve this by ordering and interpreting the reviewed literature according to the triangle concept developed by Wüstenhagen, Wolsink and Bürer (2007) as depicted in Table 1.

Thereby, this review delivers a description of the current dynamics in two core dimensions of social acceptability, socio-political acceptability and market acceptability. In terms of jurisdictions, the paper explores some of those already investigated by Ishii and Araki (2017): United States, Canada, and Australia as adopters of GM plants for cultivation, and New Zealand, Japan, and the EU as non-adopters. It explores how the latter group has responded to pressure from the emerging asynchronous regulation of GEOs, if and how there is evidence of policy changes, and what drivers and obstacles are emerging. In addition, we examine Norway and Switzerland, both part of the European region, but not members of the EU, and non-adopters in the sense described above. Emerging evidence from stakeholder and consumer research and public polls suggests that in non-adopter countries, genetic modifications resulting in smaller modifications might be perceived differently to first generation GMOs, e.g., in case of cisgenesis (reviewed by Dayé et al., 2022) and genome editing (Ishii and Araki, 2016). Over the last 3 years research activities on consumer and public views have been increasing. So this paper also provides an updated review of these studies (see Table 4). Moreover, there are recent developments indicating that the views of policy makers and food value chain stakeholder may also differ.

These developments with respect to GEOs are not only of relevance for the jurisdictions mentioned but also of relevance for other non-adopters because they are importers of agricultural commodities as well as food/feed products. Their restrictive GMO legislations and policies have already had considerable impact on agricultural and GMO policies in their trading partners—in particular exporters of agricultural products from Africa and Asia (e.g., Smyth et al., 2013). It can, therefore, be expected that indications for policy changes in these countries are very relevant for global trade and further innovations in plant breeding and will have knock-on effects on the position towards GEOs in other parts of the world (Qaim, 2020; Purnhagen and Wesseler, 2021). Beyond the jurisdictions reviewed in this paper, there are many other discussions as to if and how to accommodate in their national legislations the specifics of GEOs. The developments in the jurisdictions covered in this review will have an impact on the directions of policy development in these other countries.

Before the advent of genome editing, we have seen 30 years of public debate on GMOs in particular in the non-adopter countries, with campaigns by civil society organisations (CSOs), field trial disruptions, retailer boycotts, and Frankenfood-type headlines in media reports. Therefore, we will analyse the developments in some of our country case studies in their respective historical context. This helps to understand opportunities and challenges posed by genome editing in those jurisdictions and to suggest where policy-making and further research needs are lacking.

## Where the pressure comes from: Developments in early GMO adopting countries

### United States

Overall, publics in the United States have more favourable views of GM plants and GM food as compared to Europe (McFadden and Smyth, 2019), and there is a lower regulatory burden for marketing GM crops, which led to a rapid adoption and increase of acreage used for GM crops over the last 15 years. At present more than 175 events are cultivated on some 73 million hectares (ibid). Also, a considerable number of GM-derived food products are commercially available (see also Tables 2, 3).

**TABLE 2:** Number of GM plant events authorised for commercial cultivation per year per jurisdictions. Source: ISAAA (2021).

	Adopters^a^			So-far non-adopters^a^				
	United States^b^	Canada	Australia	Japan^c^	New Zealand	EU^d^	Norway^e^	Switzerland^f^
1992	1	—	—	—	—	—	—	—
1993	—	—	—	—	—	—	—	—
1994	8	—	—	—	—	—	—	—
1995	22	15	5	—	—	—	—	—
1996	21	15	—	—	—	—	4	—
1997	9	12	—	—	—	—	7	—
1998	13	2	—	—	—	6	—	—
1999	10	3	—	—	—	—	—	—
2000	2	1	—	—	—	—	—	—
2001	1	8	—	—	—	—	—	—
2002	7	1	2	—	—	—	—	—
2003	2	1	14	—	—	—	—	—
2004	4	1	—	13	—	—	—	—
2005	6	6	—	6	—	—	—	—
2006	2	7	3	15	—	—	—	—
2007	3	4	4	12	—	1	—	—
2008	2	4	—	10	—	—	—	—
2009	3	5	2	4	—	—	—	—
2010	2	9	—	7	—	1	—	—
2011	11	5	—	6	—	—	—	—
2012	3	9	—	8	—	—	—	—
2013	9	9	—	19	—	—	—	—
2014	19	7	3	6	—	—	—	—
2015	8	5	3	8	—	2	—	—
2016	6	7	12	9	—	—	—	—
2017	1	6	—	10	—	—	—	—
2018	2	2	4	7	—	—	—	—
2019	3	—	—	0	—	—	—	—
2020	1	—	1	1	—	—	—	—
2021	3	—	3	4	—	—	—	—
Total	184	144	56	145	0	10	11	0

a Adopter countries are countries that have authorised multiple events of GM plants and do actually cultivate them on more than 500,000 ha. Non-adopters are either countries which do not have multiple events authorized and/or do not cultivate GM-plants on noteworthy areas.

b Stacked events of registered single events are not included in the US list as they are not listed as they do not need a separate registration.

c No commercial cultivation despite approval; Japan has approved a lot of commercial GMOs for cultivation. However, commercial cultivation has been done very limited. The country has not adopted the cultivation of GMO crops even though they would have the possibility to do so.

d Commercial cultivation with one event only in some regions of the Union (Spain and Portugal), the EU had once more commercial crops approved for cultivation but approval was expired in most cases. Cultivation is exempted in some member states through Directive (EU) 2015/412 (opt out).

e Only cultivation of blue carnation for decoration purposes allowed.

f Moratorium for commercial cultivation in place since 2005.

**TABLE 3:** Number of GM plant events authorised for food and/or feed^a^ use per year per jurisdictions. Source: ISAAA (2021).

Year	Adopters^b^			So-far non-adopters^b^				
	United States	Canada	Australia	Japan	New Zealand	EU	Norway	Switzerland
1993	2	—	—	—	—	—	—	—
1994	—	3	—	—	—	1	—	—
1995	54	23	—	—	—	—	—	—
1996	40	41	—	—	—	3	—	1
1997	16	32	—	—	—	12	—	—
1998	63	8	—	—	—	9	—	—
1999	11	13	—	—	—	—	—	—
2000	8	8	9	10	—	—	—	—
2001	4	3	12	31	21	—	—	—
2002	4	2	18	6	6	9	—	1
2003	3	5	2	46	46	—	—	1
2004	16	2	3	6	6	1	—	
2005	8	14	4	17	17	3	—	1
2006	0	8	4	16	16	3	—	—
2007	10	4	3	20	20	12	—	—
2008	8	6	4	4	4	8	—	—
2009	6	6	3	6	6	10	—	—
2010	4	5	5	27	27	20	—	—
2011	12	14	5	17	17	12	—	—
2012	16	18	7	16	16	12	—	—
2013	12	6	1	38	38	23	—	—
2014	10	18	10	19	19	0	—	—
2015	20	12	2	23	23	36	—	—
2016	11	14	27	32	32	18	—	—
2017	8	12	9	21	21	20	—	—
2018	8	8	8	13	13	12	—	—
2019	4	—	—	3	6	—	—	—
2020	2	3	4	4	5	2	—	—
2021	10	5	2	10	5	—	—	—
Total	370	293	142	375	142	226	11	4

a Each authorisation is counted: combined food-feed authorisation of an event possible in some jurisdictions and some time periods count as one authorisation, separate authorisations for food and feed for the same event counts twice.

b Adopter countries are countries that have authorised multiple events of GM plants and do actually cultivate them on more than 500,000 ha. Non-adopters are either countries which do not have multiple events authorized and/or do not cultivate GM-plants on noteworthy areas.

In the United States, plants developed with biotechnology are regulated under the Coordinated Framework for the Regulation of Biotechnology (CFR). Three agencies oversee the use of biotechnology, namely USDA APHIS (United States Department of Agriculture, Animal and Plant Health Inspection Services), FDA (Food and Drug Administration) and the EPA (Environmental Protection Agency). APHIS regulates importation, interstate movement, and environmental release of certain organisms developed using genetic engineering. FDA evaluates plant-derived foods and feed products and EPA oversees products generating pesticides (e.g., Bt-Toxins) or food containing pesticide residues. APHIS regulates plants containing recombinant DNA from plant pests. Regulation by FDA is triggered by “pesticide chemical residues considered unsafe” and thereby applies to plant-incorporated protectants, such as genes for Bt toxin (Hamburger, 2019).

Motivated by a predicted increase in demand in importing countries and by simplified and less hazardous pesticide regimes, large-scale farmers growing maize and soybean were early adopters of GM plants. The papaya industry in Hawaii was even rescued by a GMO, a viral resistant papaya variety. Livestock farmers also have profited from reduced prices for feed. Civil society stakeholders, and especially environmental groups, on the other hand, opposed GM technology as fostering industrialized agriculture and monoculture mainly benefiting large multinational seed producers and because of possible environmental risks. By putting media pressure on selected value chain actors to pull out of using GM crops in their food products, they affected the strategies of certain food producers and a few crops. However, their efforts did not result in fundamental change of the market nor in a policy change. To the contrary: the US government has become an outspoken supporter of cultivation and international trading of GM crops and derived products. This included accusing the EU of violating WTO provisions by hampering market access for GM crops (reviewed in Zilberman et al., 2013).

The rapid diffusion of GM technology into agriculture and food production in the United States has not caused a profound change of the public opinion. While public views towards GM food—as identified in recent polls—are still more positive than the EU, they are nonetheless quite negative (Scott et al., 2018). In 2019/2020, 38% of US respondents agreed that GM foods were unsafe to eat. Only 27% agreed to the contrary and stated that they were safe to eat (Pew Research Center, 2020). Foods from GM plants seem to have a higher acceptability compared to foods from GM animals (Lusk et al., 2015; Runge et al., 2017). Yet, acceptability of GM food seems to increase if it has direct consumer benefits (Lusk et al., 2015; Rose et al., 2020). Comparative studies have shown that the positive effects of expected direct consumer benefits of GMOs are stronger with citizens in the US than they are with Europeans (Costa-Font et al., 2008).

Moreover, there are indications that the public appreciation of GM plants and foods in the United States has decreased slightly over the last few years; a 2016 online survey in the US revealed that 39% of respondents believed that food with GM ingredients is worse for one’s health compared to non-GM food (Pew Research Center, 2016). This number raised to 49% in 2018 (Pew Research Center, 2018). On the basis of the Pew Research Center data, it has been shown that consumer attitudes towards GMOs in the US are related with the level of polarization between political ideologies and the amount of credibility attributed to scientists in the course of this polarization (Hunt and Wald, 2020). We can thus hypothesize that a share of this decrease in appreciation during the recent years can be explained by the culture of political discourse in the US during the Trump administration—and that a calmer political culture may yet lead to an increase again.

In 2018, the US Department of Agriculture (USDA) clarified that certain types of genome-edited plants will be considered as conventional plants. In 2020 the USDA reiterated its statement not to regulate plants which could also have been obtained by conventional breeding (U.S. Department of Agriculture, 2020). Also, the FDA committed in context of its Plant and Animal Biotechnology Innovation Action Plan to pursue advances in policy priorities in order to establish a science-and-risk-based approach for product developers and to remove barriers for future innovation in plant and animal biotechnology. In 2019 these guidelines have been implemented into the new SECURE (Sustainable, Ecological, Consistent, Uniform, Responsible, Efficient) rule. The SECURE rule exempts categories of products developed through biotechnology when changes in the plant genome are: “solely introductions from sequences derived from the plant’s natural gene pool or edits from sequences which are known to correspond in the plants natural gene pool.” This also leads to an exemption of cisgenic plants from the regulation. However, the degree to which the procedures defined in the SECURE rule will promote justified public trust is a matter of contention (Wolf, 2021).

Developers can request a confirmation from APHIS that a modified plant qualifies for an exemption and is not subject to the regulations. Over the last 7 years, APHIS received some 130 requests for genome-edited varieties indicating strong interest from developers to deploy this technology (USDA-APHIS, 2021; USDA-APHIS, 2022). Since 2016 several genome-edited products have entered the market including high oleic soybean oil (Calyno™), a herbicide tolerant canola variety, and a waxy corn (Turnbull et al., 2021).

Some evidence is available from surveys on plants and derived foods produced by novel plant breeding techniques that are less “invasive” than traditional GM—including genome editing (GEOs) and cisgenesis. A choice experiment study comparing GMO apples and GEO apples showed that consumers in both France and the US do not value plant innovation by biotechnology; in both countries, consumers would purchase GMO or GEO apples only if it comes with a price discount. However, the average discount was higher with the France sample than with the American one (Marette et al., 2021). Parts of this negative attitude towards biotechnology might be explained by a distinctive effect of recency of an innovation on its social acceptability. Studies have shown that US consumers have a strong preference for crops that have been modified some time ago: The more recent the crop innovation, the less natural and beneficial and the riskier it is seen (Inbar et al., 2020). This is mirrored by the fact that consumers still prefer food derived from “conventionally” grown plants over food derived GEO plants (Caputo et al., 2020). Yet, there are also indications that US consumers value having the option to purchase them. Also, if provided with information about the benefits to themselves and the environment, the difference in acceptability between GEO and conventional plants decreases. It is thus estimated that the market share for food derived from GEO plants might exceed 15% in the near future (Caputo et al., 2020).

There is some awareness in broader publics of novel plant breeding techniques, but a recent study showed that about a third of US adults have never heard or read anything about genome-edited food (Peters, 2021). Also, discourse analyses in social media showed that very often, GEOs are conflated with GMOs (Walker and Malson, 2020). States and regions where the agroeconomy is visible and present in the public discourse tend to have both a higher awareness of the differentiation between GMOs and GEOs and a higher appreciation of the potential environmental benefits of their use with crops (Wirz et al., 2021). While non-government organisations (NGOs) which hold critical views toward GMOs are lobbying to have GEOs and cisgenic plants and food put into the same regulatory categories as GMOs (Ishii and Araki, 2016; Smyth, 2019), this does not seem to have a relevant impact on the behaviour of value chain actors and policy development.

### Canada

Canada’s legislation is distinctively different to any other legislation frameworks as it is triggered by novelty (in terms of plant traits) and thereby potentially applies to all plant varieties regardless of the breeding technique used. If a new plant trait is classified as novel the same requirements for pre-market safety assessment and approval apply. All products are considered on a case-by-case basis for novelty (Smyth, 2019). However, Canada recently released a draft guidance for Part V of its Seeds Act. Part V deals with regulatory requirements for both the confined and unconfined environmental “release of seed.” The draft guidance states that “gene editing techniques can introduce genetic changes that are comparable to conventional breeding outcomes, and will also qualify for an exemption.” Canada is willing to exempt categories of GEOs from its regulation as long as they are comparable to conventional breed plants. There is guidance available for developers and breeders as to what constitutes novelty, and if molecular genetic methods are used, the government encourages developers to seek feedback from the Canadian Food Inspection Agency and Health Canada. Still, research has shown that some stakeholders in plant breeding and plant science perceive these regulations are hindrances of their research and innovation activities (Smyth et al., 2020).

Like in the US, public views on GM plants and GM food in Canada are more favourable than in Europe (McFadden and Smyth, 2019). Commercialisation of GM plants in Canada started early and reached 12.5 million hectares in 2019 with adoption rates ranging between 95 and 100% for their main agricultural crops, i.e., soybean, canola, and maize (ISAAA, 2019) (see also Tables 2, 3 which reveal similar dynamics as in the United States). There are no mandatory labelling or traceability requirements in place for GM food and feed, so uptake by food and feed processers is difficult to estimate. Considering the high adoption rate in North America most processed food and feed products originating from this region can be expected to contain GM ingredients.

Similar to the United States, however there are indications that public attitudes have become more negative over the last few years: a 2016 survey in Canada found that 62% of the respondents agreed that they would always opt for a non-GM food over GM. Only 26% expressed being comfortable with eating GM foods, and 38% stated to be not comfortable (The Strategic Counsel, 2016). Between October 2019 and March 2020, 39% of the Canadian respondents agreed that GM foods are unsafe to eat and 27% agreed that they are safe to eat (Pew Research Center, 2020). Food safety appears to be an important concern of Canadian consumers, and it has been shown that this overshadows their acceptance of GMO applications (Goddard et al., 2018). However, it has also been shown that this concerns are set aside when there is a price or a nutritional benefit of the GMO product compared to a “conventional” product (Macall et al., 2021).

Some evidence is also available from studies focusing on gene editing. While certain civil society organisations hold critical views toward GMOs, this does not seem to have a relevant impact on the behaviour of value chain actors as well as public and consumer views. A survey study by Vasquez Arreaga (2020) that invited Canadian consumers to compare descriptions of GMO and GEO foods yielded 15% more positive responses for GEO foods. Benefits—both benefits to the consumer and for the environment—operated as the main drivers for positive responses. If framed as “more natural” than GM transgenic plants, consumer acceptance increases for both GEOs and cisgenic plants (Muringai et al., 2020). Further, cultural values have been shown to be a potential lever in order to increase consumer acceptance of GEOs (Yang and Hobbs, 2020).

### Australia

In Australia, market approvals of GM crops progressed at a slower pace compared to United States and Canada (Table 2). Still, it resulted in widespread planting of several GM crops (e.g., cotton, canola and safflower) over the last decade. Australia’s approach from early in the development of regulations for GMOs has had a focus on the product of the targeted event/gene, and not on the process in which it was delivered. Regulations include the Gene Technology Act 2000 (GT Act) and GT Regulations 2001 which provide definitions of GMOs and guides to exclusions and inclusions to what is regulated (Thygesen, 2019). In 2016, a review of the regulation clarified the situation regarding gene editing. Edited plants or animals containing edits but with no guide or extra DNA, classified as SDN1 organisms (SDN: site-directed nuclease), have been given a “non-regulated” status in Australia. SDN1 events were given this non-regulated status, as the product cannot be distinguished from those naturally occurring DNA changes.

Australia consists of six states, and ten territories, yet gene technologies are regulated under a national/federal regulatory scheme. All work with GMOs (i.e., import, research, commercial release, manufacture, or production) is prohibited unless the entity is licensed or falls under an exception. Several states have overridden national decisions in enforce a state-wide ban on GMOs (e.g. South Australia), with several of these decisions now reversed, while Tasmania still has a broad prohibition in place. The planting of GM crops is regulated by the Office of the Gene Technology Regulator (OGTR). The regulation of GM use in foods is covered by Food Standards Australia New Zealand (FSANZ) who approve, or not, all foods based on safety assessments before they can be sold in Australia and New Zealand. This group is currently deliberating on the status of foods having gene edits, a process that started in 2017 (Kelly, 2019). Their long-awaited decision could well affect the previous decision by the Federal Government to exempt organisms with SDN1 events from regulations for cultivating organisms. It also affects the situation in New Zealand.

This process, involving the OGTR and FSANZ, appears to be well trusted in Australia. A large, representative study of Australian citizens that covered the years 2004–2012 (*n* = 8,821, almost equally distributed over 9 years) used a 11-point scale (ranging from 0 = “not at all comfortable” to 10 = “very comfortable”) to measure attitude towards GMO plants and animals. It showed that the public leant more positive to GM plants (*mean* ≈ 4.14) than to GM animals (*mean* ≈ 2.95). Still, both figures are clearly in the negative half of the spectrum (Marques et al., 2015). It was also shown that the positive attitude toward GMO food was significantly associated with a high trust in scientists and regulators. Environmental groups acting as ‘watchdogs’ were trusted less. Further, the study showed that the attitudes towards GM food remained rather stable over the 9 years covered. There were smaller ups and downs, but these were related to media debates and did not indicate a growing or shrinking rate of acceptance.

A more recent study (Batalha et al., 2021) explored whether consumers held different attitudes towards classical undirected mutagenesis, GMOs, and GEOs. Study participants (*n* = 114) felt that mutagenesis, introduced as “process of exposing seeds to chemicals or atomic radiation in order to generate mutants with desirable traits to be bred with other cultivars” (Batalha et al., 2021, Appendix A), was the riskiest breeding technique. GMOs were perceived to be less risky, but still riskier than GEOs, which ranked close to plants derived from conventional breeding. This last finding indicates that compared to other countries, public opinion in Australia is rather positive towards GEOs, an assessment that comparative studies confirm (Shew et al., 2018).

Despite this rather positive public perception, all GMO plantings so far have been of non-food crops. However, the decision to “de-regulate” edited SDN1 organisms could mean rapid deployment of new varieties and products that could be traded freely in Australia, and potentially to export markets.

## How so-far non-adopters of GM plants have responded

### Japan, deciding to open-up

In the mid-1990s, social controversy erupted in Japan over foods derived from GM crops (Yamaguchi and Suda, 2010), with the media emphasizing the unknown risks of GM foods (Shineha et al., 2008) and consumer advocacy groups organizing boycotts. GMOs were socially stigmatized to a degree that deterred the interest of seed producers (Tano, 2015) and damaged the credibility of scientists (Yoshida, 2015). These social phenomena laid the groundwork for the current regulatory regime (Ishii, 2019).

In Japan the commercialization of transgenic crops and food products requires specific approvals. Four ministries are involved in the regulatory framework: the Ministry of Agriculture, Forestry and Fisheries (MAFF); the Ministry of Health, Labour and Welfare (MHLW); The Ministry of Environment (MOE); and the Ministry of Education, Culture, Sports, Science and Technology (MEXT). Food and feed safety risk assessments for MHLW and MAFF are carried out by the Food Safety Commission (FSC), an independent risk assessment body. When Japan ratified the Cartagena Protocol on Biosafety in 2003, the government adopted the “Act Concerning the Conservation and Sustainable Use of Biological Diversity through Regulations on the Use of Living Modified Organisms” also called the “Cartagena Act” (Ministry of Justice, 2003). The Act in Article 2 (2) defines LMO as:

…*an organism that possesses nucleic acid, or a replicated product thereof, obtained through use of any of the following technologies: (i) Those technologies as stipulated in the ordinance of the competent ministries, for the processing of nucleic acid extracellularly (ii) Those technologies, as stipulated in the ordinance of the competent ministries, for fusing the cells of organisms belonging to different taxonomical families*.

Some local governments established ordinances restricting commercial plantings of genetically modified crops. Though the number of approved events are relatively high (see Tables 2, 3), these multiple layers of statutory requirements send signals to stakeholders that Japan takes a “precautionary” stance to the commercial planting of gene modified plants (The Law Library of Congress, 2014), thus discouraging industries and producers to use transgenic seeds for commercial purposes.

In 2019, however, the Japanese government issued a ruling on the interpretation of the Cartagena law, under which genome editing techniques that do not leave extracellularly processed nucleic acids will not be subject to regulation. Also excluded are processes using the nucleic acid of an organism belonging to the same species as that of the target organism or the nucleic acid of an organism belonging to a species that exchanges nucleic acid with the species of the target organism (Ministry of Agriculture, Forestry and Fisheries, 2018).

On 8 February 2019 (Notification No. 1902081), the MAFF was advised by the expert committee that:

…(i) any organism that has inserted extracellularly processed nucleic acid (including RNA) is regarded as a living modified organism (LMO), even those obtained by using genome editing technologies, and is subject to the regulations stipulated in the Cartagena Act, in principle, (ii) such organisms are subject to the Cartagena Act unless complete removal of the inserted nucleic acid (including RNA), or its replicated product, is confirmed, and (iii) when using organisms obtained through genome editing technologies, applicants are requested to submit information to the competent government agencies, even when the organisms are not subject to the Cartagena Protocol.

Based on these legal definitions, some genome-edited crops, such as foods derived via SDN1-type events, are exempt from regulation. Following up on the notification related to the Cartagena Act, the Food Sanitation Commission of Pharmaceutical Affairs and Food Sanitation Council published guidance on 27 March 2019 indicating that insertion of one to a few bases will not be regarded as producing LMOs. In April 2021 MAFF and MHLW amended the handling procedures for food and feed additive products, indicating that no prior consultation is needed for crosses of genome-edited varieties previously notified to MAFF with conventional varieties or for other previously reported genome-edited varieties and GM products which have obtained feed safety approval.

Studies of stakeholder views of genome-edited foods are somewhat limited. Among the few studies that exist, the report published by the Ministry of Agriculture, Forestry and Fisheries (2018) suggests that stakeholders including scientists, producers, seed and seedling companies, distributors and retailers see three issues as key: establishing clear and uniform regulations in place of the current patchwork of national and local standards; developing crops and foods that will be perceived as having high added value to consumers (such as foods with particular health benefits); and improving social acceptance of GM foods (see also Jorasch, 2020).

Earlier studies indicated that publics are sceptical of GM crops. A study carried out in June 2001, for instance, showed that 80% of the survey respondents (*n* = 400) would not purchase a GM food product even if it was substantially cheaper than a conventional product (McCluskey et al., 2003). More recently, Kato-Nitta et al. (2019) found that although lay publics tended to have more favourable attitudes toward genome editing than toward genetic modification, such differences were much smaller than the differences between attitudes towards conventional breeding and genetic modification. Also, a study amongst university students (*n* = 180) showed that the willingness to purchase genome-edited food was 24%. However, after more information about genome editing technologies was given to respondents the willingness-to-pay (WTP) increased to 41% (Farid et al., 2020).

In an attempt to understand Japanese consumers’ experience of genome editing technologies, Otsuka (2021) examined the correlation between perceived naturalness and perceived safety of various breeding technologies. Consumers were asked to rank scores for foods derived from crops developed by five breeding technologies: epigenome editing, genome editing, genetic modification, mutagenesis by chemicals or irradiation, and crossing as in conventional breeding. Conventional breeding was seen as the safest, followed by mutagenesis, epigenome editing, and genome editing; genetic modification was last. This study suggests that consumers perceive GE crops as less natural, and more similar to GM food, than those with mutagenesis achieved by chemicals or irradiation. This mirrors an earlier study on the perceived differences between transgenic and cisgenic crops (Kronberger et al., 2014).

A report published by the National Institute of Science and Technology Policy compared consumer attitudes to various technologies such as hydrogen energy, robots, autonomous cars, etc. This indicated that consumer responses to GE foods are similar to responses to GM food (Hosotsubo et al., 2020). Ishii (2017) speculates that GM food and GE food are “bracketed” in the same food category, and that a segment of consumers might reject the use of GE foods. On the other hand, in a study of the willingness to purchase apples altered by the use of agrobacterium-mediated transformation, Saito et al. (2017) point out that providing information about the ecological benefits of planting those apples will make some consumers more receptive to GM apples.

While government policy on labelling requirements for genome-edited foods is somewhat tentative, consumers have started to call for a regulation of the cultivation of all genome-edited crops, as well as for safety reviews and labelling of all genome-edited foods so that consumers can make informed choices (Mainichi Shinbun, 2018). On the other hand, anecdotal evidence suggests that genome-edited foods are positively received in some market segments. Adopting marketing strategies such as direct-to-consumer selling and crowdfunding campaigns for commercialization of genome-edited fish, companies have been reaching out to consumers in a specific segment of the market. The shaping of full social acceptability of genome-edited foods is yet to be observed, but the presence of genome-edited foods is becoming stronger.

### New Zealand, not opening up

As of 2022 New Zealand (NZ) did not authorise any GM crops for cultivation (see also Table 2) and takes a hard line in its interpretation that there is no difference between a GMO and a GEO. This is due to an early decision (1996) to regulate organisms based on the process that was used to generate a “new organism” (i.e., the *in vitro* manipulation of DNA), rather than what the product of any *in vitro* event may contain. More specifically relating to regulation of organisms as a result of gene editing, NZ was one of the first countries to amend their legislation to distinguish, and regulate differently, genome-edited plants from those bred by conventional mutagenesis (Fritsche et al., 2018).

The regulations controlling GMOs and GEOs are contained in the 1996 Hazardous Substances and New Organisms Act (HSNO) and administered by the Environmental Protection Authority (NZ EPA). They are one of the more comprehensive in the world, with strict standards for approval assessment (Kershen 2015). In September 2016 these regulations were amended so as to include genome editing (new forms of mutagenesis performed *in vitro*), but allow plants created by older forms of mutagenesis (pre-1998). This implies that novel plants created by new breeding techniques, even those without foreign DNA, still fall under the regulations as a GMO (Ishii and Araki, 2017).

In assessing the safety of a GMO and GEO, the NZ EPA must consider whether the benefits of the new organism outweigh the risks including the impact that the novel plant may have on the Māori culture and traditions, especially with regards to their valued fauna and flora, ancestral lands, water, sacred places and treasured things (Hudson et al., 2019). Using this framework, several GMOs have been released (which are vaccines and medical treatments). No crop has satisfied this framework, and therefore there has been no approval for release.

Food safety regulations in NZ are controlled by a joint Australian-New Zealand authority, FSANZ. This has led to the perplexing situation where-by some plant products are considered safe in NZ to eat, but not considered safe to grow. Golden rice is one such example; in 2017 FSANZ released a decision deeming Golden rice (strain GR2E), “…food derived from GR2E is considered to be as safe for human consumption as food derived from conventional rice varieties” (FSANZ, 2017).

Without any GMO or GEO being grown, stakeholder views in NZ are hard to judge. Federated Farmers (an advocacy group for NZ farmers) have been supportive of science-led evidence (provided by groups such as Universities and the NGO Life Sciences Network) on GMO and GEO safety in various court cases. This support reflects concerns of NZ farmers and growers that the technologies and plant varieties they use should be the most current, allowing them to compete globally. This advocacy by science and industry groups has had mixed success, with some regional councils effectively placing bans on GMOs/GEOs, even if they were to be approved by the very precautionary nationally enforced HSNO Act.

The NZ Government has stated that a cautious approach to genome editing is appropriate because as an exporter of premium food products NZ needs to protect market perceptions of purity and safety (The New Zealand Government, 2016). Even though the bulk of NZ exports are to countries which currently grow GMOs and allow un-regulated growth of GEOs (i.e., less than 8% of NZ exports are to the EU and the United Kingdom) consumers of premium branded NZ fresh food are thought to value the “100% Pure” branding exercise (Kaefer, 2016). This position has remained unchanged in spite of studies showing that the vast majority of value chain stakeholders were convinced that no such cross-over effect would occur and that growing GM/GE crops in NZ would not impact export of non-GM products (Knight et al., 2005b). The fear that there would be a negative impact on the tourism sector has also been proven to lack justification (Knight et al., 2013). In contrast, the NZ Government is also supportive of technologies that help diversify the primary sector, to safeguard against downturns in one industry (induced by the market, climate change, or new diseases).

Despite market approvals for a considerable number of products which include GM ingredients (see Table 3), no fresh GM/GE plant products are available in NZ for consumption, although the Golden Rice decision, by FSANZ, is as close to a whole food GMO approved for consumption if available. Fruit stall experiments simulating real purchasing behaviour in New Zealand showed that consumers were willing to buy GM fruits if they had a clear consumer benefit (Knight et al., 2005a; Mather et al., 2012). Other GMO processed products are in NZ supermarkets but not widely discussed, e.g., the plant-based meat product, the Impossible Burger, is now being sold in NZ. Māori have been significant contributors to the debates on GM in New Zealand and have insisted on their values having specific recognition on GMOs/GEOs (Roberts and Fairweather, 2004; Everett-Hincks and Henaghan, 2019; Hudson et al., 2019). Their core cultural values, including ancestry (whakapapa) and guardianship (Kaitiakitanga), have been analysed. If these values were enhanced by genome editing then it could allow more favourable discussion on a genome editing approach (Hudson et al., 2019). This more dynamic approach to specific uses or types of uses could then be approved on a case-by-case basis, which is not supported by NZs current legislation.

### Norway, considering opening up

Norway is not part of the European Union, but a member of the European Economic Area (EEA). According to the EEA agreement, the EU-harmonised GM legislation also applies to Norway but allows for additional legal measures. This entitles the Norwegian Parliament the rights to enact a more comprehensive legislative framework and thereby to permanently restrict or prohibit a GMO that has been authorised EU-wide for other reasons than those laid down in the EU regulation (health and safety risks). Accordingly, the Norwegian Gene Technology Act (NGTA) of 1993 foresees the mandatory requirement to assess each GMO and to ban it for the Norwegian territory if the GMO does not meet the criteria (Miljøverndepartementet, 1993). In addition to the health and environmental safety criteria specified by the EU, the NGTA also requires the assessment of criteria in three non-safety categories: benefits to society, effects on sustainable developments, and, whether the production and use will take place in an ethically and socially justifiable way. The geographical scope of the sustainability criterion also includes impacts in the countries of cultivation and/or production, thereby also incorporating environmental effects also outside Norwegian territory (Kvakkestad and Vatn, 2008; Turnbull et al., 2021).

So far, no plant has been authorised neither for cultivation in Norway, nor for food/feed use. The only GM plants authorised were imports of carnation events with modified blossom colour. The ten GM plants authorised for cultivation in the EU (see also Table 2) have been banned in Norway. This situation can be understood as reflecting both the absence of interesting products for the Norwegian market and the views of broader publics. First generation of GM crops, mainly varieties of soybean and maize, had little to offer to Norwegian consumers. Rather, they were perceived as a potential threat, adding to what was perceived in Norway as a major environmental health problem: antibiotic resistance. This helped critical stakeholders to mobilise against GM crops.

In line with this development public surveys and consumer studies from the late 1990s and early 2000s revealed a strong opposition to GMOs (Chern and Rickertsen, 2001). However, the data also indicated a weaker opposition if GM plants came with environmental benefits, e.g. reduced pesticides, or improved nutritional value. These views seem to have been relatively stable over time. A follow-up study conducted in 2017 using same and similar questions showed fewer respondents willing to avoid GM food compared to the 2001 study (Rickertsen et al., 2017). Another study by Bugge (2020) compared changes between 2017 and 2020. There were only small differences in most of the questionnaire items used (role for world’s food supply, impacts on nature and ecosystem in general and on pesticide use in particular, human and animal health risks, role for industrial agriculture). Interestingly, however, the group positive on selling such products in Norwegian stores grew from 15% to 24%. When confronted with examples of GM food approved for the US market, blight resistant potato received the highest acceptability (60%) compared to fast growing salmon (20%).

Against this backdrop of a relatively stable negative public opinion, in January 2018 the Norwegian Biotechnology Advisory Board initiated a public debate on revising the NGTA in light of genome editing and other novel breeding technologies (NBAB, 2018b). Following a series of consultations with stakeholder groups, they developed a proposal for a tiered system for deliberate release of GMOs that foresees that organisms with changes that can exist naturally or that can be achieved using conventional breeding methods (Tier 1) are no longer required to pass the assessment and approval procedure. Instead, notification to the authorities and subsequent confirmation is considered sufficient. With respect to labelling, the opinions of the Board diverged, with about halve of the Board members suggesting to exempt Tier 1 from labelling requirements at all. In order to qualify for Tier 1 a genome-edited variety would “be possible to make using non-regulated methods,” e.g., point mutations (therefore similar to SDN1), and would require evidence of absence of off-target mutations (NBAB, 2018a).

This proposal was intended as input into both the national debate and the debate on revising the EU GM legislation in light of the CJEU ruling. While the review of GTA is still ongoing, the Norwegian government is expected to present a proposal for amendment by the end of 2022 (Kongelige Klima- og Miljødepartementet, 2020; Kongelige Klima- og Miljødepartementet, 2021).

Stakeholder responses seem to suggest that parts of the food value chain would prefer regulatory amendments, which would allow certain types of genome editing to be used by breeders in Norway. This is a remarkable change of position, as theses stakeholders so far pursued a strict no-GMO policy. One large interest organisation of 17 agricultural cooperatives comprising breeders, farmers and food processors publicly declared that they started a review process of their GMO policy (Norsk Landbrukssamvirke, 2021). Strong support is coming from certain plant and animal breeders who are eager to use this technology, e.g., for major Norwegian pest problems such as late blight in potatoes (Graminor, 2021; Norsvin, Geno, and AquaGen, 2021).

A parallel survey showed that Norwegian consumers seem to be more positive towards GEOs if they had tangible social or environmental benefits, e.g., by reducing pesticide use, crop losses, climate adoption, improved nutritional value etc. (GENEinnovate, 2020) A majority of respondents (*n* = 2016) were in favour of using genome editing in organic food production if it allowed cultivation without pesticides. 66% of the respondents were very or somewhat positive towards the idea to use genome editing in order to reduce pesticide use and crop loss with the example of blight resistant potato; only 10% were very or somewhat positive towards using it to create a salmon with more brightly pink coloured meat. Genome-edited plants developed within Norway and for domestic products are perceived positively by 45% of respondents (23% respondents had a negative perception); in comparison, GMOs currently on the international markets and developed by international companies were positively perceived by only 20% (45% negative perception) (ibid).

Still, a majority of 60%–70% respondents were worried about risks to health and the environment. While respondents did not seem to differentiate in terms of naturalness between GE and GM, they would not be willing to pay very much for avoiding GE foods. Also, the above-mentioned study of the NBAB has been criticized for being biased. Critics stated that the initiative’s privileging of technological matters and its framing of the discussion in economic terms would have “skewed the proposal in a way that reduced broader societal concerns to technological definitions and marginalized discussion of the social, cultural, and ethical issues raised by new gene technologies” (Kjeldaas et al., 2021).

Mandatory labelling was considered by respondents to be very important in case of GM food products (Rickertsen et al., 2017; Bugge, 2020) and this also seems to apply for genome-edited food (GENEinnovate, 2020). Approximately 70% of respondents think that such a label should distinguish between genome editing and classical genetic modification and more than 80% stated that it should contain information about the trait and the purpose for making it (ibid).

### Switzerland, considering opening up

Despite its location in the centre of continental Europe, Switzerland is neither a member of the European Union nor of the European Economic Area. It is relationship to the EU is governed by an ever-increasing number of sectoral bilateral agreements, also relevant for Swiss GMO legislation.

GMOs authorised in the EU still need authorisation under the Swiss Federal Act on Non-Human Gene Technology (GTG, Bundesversammlung der Schweizerischen Eidgenossenschaft, 2003). Triggered by prevailing negative views among publics and stakeholders (Bonfadelli and Dahinden, 2002; Siegrist, 2003; Gaskell et al., 2004; Einsele, 2007; Bonfadelli and Meier, 2010; Connor and Siegrist, 2010) which perceive GMOs as associated with health and environmental risks, as being morally problematic, and as lacking benefits, a national referendum to temporarily ban GM plants from Swiss fields was approved in 2005. This ban was subsequently renewed several times.

This development has to be seen against the backdrop of policy changes in Swiss agriculture.

In the late 1990s, Switzerland adjusted its agricultural policy towards de-intensification and sustainability. Integrated farming practices were increasingly adopted and within 20 years organic farming grew from 2% to 16% of total arable land. The share of organic retail sales reached more than 10% in 2019, the second highest market share worldwide (Aerni, 2010; Willer et al., 2022).

Also, in the 1990s, GMOs became a prominent media topic and symbol for industrialised farming which helped to alert both stakeholders and broader publics. The Swiss Alliance GMO-free (Schweizer Allianz Gentechnikfrei, SAG), a civil society organisation active from 1990 onwards, became the national hub for GMO-critical stakeholders. Its broad and increasing membership includes organic farming and small farmer’s associations, breeding companies, and organisations campaigning on environmental, consumer, nature protection, or animal protection topics.

Resistance from both the public and various stakeholders was reflected in restrictive regulatory measures. As a consequence of these measures, as of January 2022 no GM plants are authorised for cultivation (see also Table 2). Three GM maize and one GM soybean event were authorised for food use and another six events authorised in the EU were declared tolerated in food up to 0.5 percent per ingredient (see also Table 3). Acknowledging the dependency of Swiss animal farming on imported feed, some 30 events were authorised for feed use and another 40 events tolerated (Bundesamt für Landwirtschaft, 2014; Amt für Umwelt, 2016; Eidgenössisches Department des Inneren, 2020; BLV, 2022). Hence, a few GM plants could, in principle, legally be used for food and a large range is available for feed purposes. Still, in response to public concerns and campaigning CSOs virtually no GM food products can be purchased in Swiss retail stores. Feed producers also strive not to provide GM feed for farm animals, partly because this is requested for organic farming and private quality labels like Coop Naturafarm, TerraSuisse, or QM-Schweizer Fleisch (Akademie der Naturwissenschaften Schweiz, 2013; IP-SUISSE, 2022).

In 2016, several governmental and non-governmental organisations started to explore the opportunities and challenges for the economy and for the regulatory system associated with Novel Plant Breeding Techniques (NPBTs), in particular with genome editing. A Swiss technology assessment study conducted in 2018/2019 identified and described the polarised views on genome editing as very similar to conventional GMOs (Spök and Hammer, 2019). Plant scientists, parts of the breeding community, and biotech industry highlighted the technical and economic potential. In addition, the Federal Office for Agriculture assigned great potential to NPBTs in its long-term strategy 2050 (Bundesamt für Landwirtschaft, 2016). Organic farmers and the GMO critical SAG alliance, in contrast, portrayed GE as just another variant of GMOs and considered the Swiss GM legislation as fully appropriate. Domestic retail chains, however, remained rather silent.

Between these opposite poles, some stakeholders were more nuanced but still very clear. As the umbrella association of Swiss farmers (Schweizer Bauerverband SBV) argued:

„Transparency and credibility are key, consumer opinion is important. As long as society equates NPBTs with GMOs, products made with these methods have no chance on the market. And as long as there are no market opportunities, agriculture should produce NPBT-free.” (Schweizer Bauernverband, 2017, p. 17; transl. by authors)

In 2019, approximately 80% of the Swiss Members of Parliament were in favour of extending the ban on cultivation of GM plants and a majority of delegates wanted to strictly regulate genome editing (Schweizer Allianz Gentechfrei, 2020). Consequently, the Federal Council, the larger of the two chambers of the Swiss Parliament, decided to prolong the moratorium for another 5 years in 2021 (Schweizerische Eidgenossenschaft, 2021). However, in early 2021 an informal network of value chain actors led by a core group including large retailers started to explore views and coordinate strategies on the topic. In the final months of 2021, they established themselves as a formal alliance “Sorten für Morgen” (Varieties for Tomorrow). The alliance brought together Swiss food retailers, covering almost 80% of the Swiss market, food and producer associations, the Swiss association for integrated farming (IP Suisse) with almost 19,000 members (of a total of some 50,000 farmers active), a seed association, and a group of breeders. Repeatedly, they issued media releases calling for more openness towards genome editing (Sorten für Morgen, 2021a; Sorten für Morgen, 2021b; Sorten für Morgen, 2022). In parallel, studies in Switzerland confirmed what had been found elsewhere: that the acceptability of GM and genome editing techniques increased when they are associated with direct environmental benefits, i.e., a reduction in pesticide use (Saleh et al., 2021).

Being aware of these recent initiatives of major food value chain stakeholders, the smaller chamber of the Swiss parliament, the Council of States, suggested in December 2021 to exclude from the moratorium GEOs which do not contain DNA from non-crossable species from the moratorium. This would exclude cisgenic and genome-edited plants of SDN1/SDN2-type alike (Bundesversammlung der Schweizerischen Eidgenossenschaft, 2021).

Brought under pressure by these activities, the association of conventional farmers (SVB) decided in January 2022 to drop generic opposition to GMOs and acknowledge that certain types of genome-edited plants could be of value for Swiss agriculture (Häne, 2022). They did not, however, follow the view of the Federal Council of States. Rather, they argued that the Swiss government should explore possible scenarios and elaborate a proposal by a firm and legally agreed deadline. This would allow for more time during which the moratorium would apply also to genome-edited plants. In March 2022, the National Council followed this proposal, assigning the Council of States with the task to develop a proposal for regulation amendment by 2024 (Bundesversammlung der Schweizerischen Eidgenossenschaft, 2022). For the first time in 30 years of GMO policy development, these recent developments seem to indicate an opportunity for a policy change.

### The European Union, opening–up or remaining closed?

The European Union (EU) as supra-national entity is a unique case: aimed at developing and maintaining a smoothly working internal market by harmonising legislation, it developed into a broader project of economic and political integration. Harmonisation and integration, however, do not (yet) imply centralised decision-making. In contrast, even in legally harmonised policy areas the EU is operating as a multilevel system where a considerable share of decision-making power remains with Member States. So-called “Implementing Decisions” are legally binding and directly applicable in all Member States, but they usually have to pass a vote of Member States representatives in one of the Standing Committees or of national ministers in the European Council. Development and amendment of harmonised EU legislation is a very time-consuming process, as it not only involves all Member States via the European Council but also the European Parliament. Furthermore, such processes require several rounds for commenting and revision. Also, most larger legislation projects in the EU aim to involve citizens as well as stakeholders in the debates in order ensure that all views are considered and the result is well-balanced. These characteristics are important to consider when analysing EU policy developments and anticipating future scenarios.

Since the early 1990s the process of establishing and further developing a harmonised EU legislation on GMOs has triggered and—in turn—has been strongly influenced by opposition and campaigns of an influential alliance of civil society organisations and parts of the farming community (Schurman and Munro, 2010). In some Member States, this alliance became broader and more powerful as it also succeeded to get retailers, food producers, green parties, and media on board. Consequently, in Member States such as Austria, Hungary, Italy, and Greece, rejection of GMOs as crops and in food became the dominant and institutionalised position. This motived the introduction of national legislations that effectively act as barriers for GM plant cultivation. These countries began to advocate an even stricter legislation at the EU level, too (Tosun, 2014; Stephan 2015).

The long-lasting narrative of GM crops as posing a risk to health and environment, and the absence of clear advantages of first generation of GM crops outside the farming community was also reflected in public surveys. Between 1991 and 2010, consumers became more averse to GM products. In 2005, the majority of the respondents in a Eurobarometer survey described GM foods as morally not acceptable, not useful, and risky; research in this direction should not be encouraged (European Commission, 2006). In 2010, two thirds of EU consumers were very (27%) or fairly (39%) worried about GMOs found in food or drinks, putting GMOs on rank five in a ranking of perceived food risks (European Commission, 2010). However, studies that focused less on the stated preferences but on actual purchase behaviour indicated that this opposition was stronger on the discursive level than on the level of practice. In actual purchasing decisions, consumers appeared to be more open towards GM food than when asked for their preferences (Mather et al., 2012; Sleenhoff and Osseweijer, 2013).

The resulting harmonised legislation on GMOs and derived food/feed products require a risk assessment by the European Food Safety Authority (EFSA) together with Member States’ national Competent Authorities. Authorization for marketing or cultivation requires a majority vote of EU Member States in the Standing Committee of Plant, Animal, Food and Feed (ScoPAFF). However, this regulatory regime provided considerable leeway and options for Member States to delay or opt out of market approvals granted at the EC level. At least with respect to cultivation of GM crops the EU failed to establish a common market. Commercial cultivation is ongoing with some 100,000 hectares in Spain and Portugal. Authorisation of GM food and feed have been facing less resistance during and after market approval. Over time, 10 GM events have been authorised for cultivation, and 226 for use in/as food and/or feed (see Tables 2, 3). Currently, only one event (MON810) has the approval for cultivation in the EU. In some Member States, such as Austria, France, Germany, Hungary, however, the use of GM food was effectively undermined by other means: pressure by campaigning civil society organisations on food retailers and processors not to use GM ingredients, initiatives to establish GM-free regions, or by strengthening the role of organic farming (Bernauer and Meins 2003; Weimer 2019; Seifert, 2021).

In an attempt to overcome this difficult situation, in 2015, Member States were allowed to opt-out from centralised authorisation for cultivation of GM crops by the EC—effectively granting them the possibility to ban crops on other than health or environmental safety reasons (European Parliament and Council, 2015). However, in the absence of a significant effect of this most recent measure, the EU system was criticised by some as not fit for purpose or even as failed (Dabrowska-Klosinska, 2022). Still, it was not clear how to proceed.

Against this backdrop, novel developments in molecular plant breeding techniques received particular attention. In 2006 a paper published in *Nature Biotechnology* triggered a debate whether cisgenic plants should be regulated the same way as other types of transgenic events (Schouten et al., 2006; Jacobsen and Schouten, 2008). The authors proposed to exclude them from the EU Directive 2001/18/EC. This was justified as cisgenic organisms pose less risk than transgenic organisms—a view also shared by EFSA in their 2012 opinion (European Food Safety Authority, 2012). In the same year, an EU level Expert Group concluded that cisgenic and intragenic plants fall under the harmonized EU legislation (European Parliamentary Research Service, 2020).

A similar question came up with genome-edited plants of SDN1- and SDN2-types. Promoters of this view were for some time optimistic that these types of genetic changes will be considered same or similar than conventional mutagenesis which is excluded from GM legislation. On 25 July 2018, however, the European Court of Justice ruled that induced mutagenesis—regardless if resulting in very minor changes—cannot be exempted from EU GM legislation in the same way as conventional undirected mutagenesis—essentially because of the limited experience with this method which did not exist at the time of the regulation was established (European Court of Justice, 2018). The prevailing interpretation of the ruling by the European Commission and legal scholars is that all types of genome editing are regulated the same way as transgenic organisms - including the need for developers to provide unique identifiers, the requirements for pre-market risk assessment and for labelling (European Council 2019; European Commission 2021b; extensive list of references provided by Dederer and Hamburger 2022).^1^

The Council asked the European Commission to conduct a study on the impact of the CJEU ruling of 2018 (European Council, 2019). The study took into account the state-of-the-art knowledge, ethics and the views of the EU countries and stakeholders. In 2021 this study concluded that the EU legislation is not fit for purpose for some new genomic techniques (NGTs). It highlighted the possible role of NGTs in the transformation towards a more sustainable agri-food system outlined in the European Green Deal and the Farm to Fork and biodiversity strategies. Besides enforcement and implementation challenges for traceability and labelling, the study also diagnosed risk assessment requirements for GMO as disproportionate types of NGT resembling changes which can also be achieved by classical mutagenesis in conventional breeding (European Commission, 2021b). On this basis, the EC suggested a revision and presented a roadmap including citizen, stakeholder and Member State consultations in 2022. The EC plans to develop a proposal by mid-2023. This proposal should also allow considering the possible contributions of these plants to the above-mentioned agro-feed and environmental policy objectives (European Commission, 2021a). The later aspect is perhaps the most interesting one as it indicates a significant shift in the legislation.

This consultation process has just started and it is too early to anticipate further steps. Still, a few observations relevant for both the process and the outcome can be made. First, there are indications of policy changes in some EU Member States indicating that the Member State’s block of GMO opponents is crumbling. While the so far GMO opponents Austria, Croatia, Cyprus, Greece, and Lithuania are in favour of treating all genome-edited organisms as GMOs—this is not true for Italy, Hungary, and the Slovak Republic (EURACTIV, 2019; European Commission, 2020b, Replies from Member States). The views of other Member States are still not clear, of particular importance will be Germany and France—both with internally conflicting views of their national ministries.

Second, evidence from surveys and consumer studies suggests that publics and consumers, if compared to first generation GM crops and derived food, might be more open towards techniques that create what they perceive to be smaller modifications in the genome and the resulting plant as “more natural”. Confirming earlier studies (Mielby et al., 2013), reanalyses of the 2010 Eurobarometer data showed that Europeans differentiate between trans- and cisgenic plants (Hudson et al., 2015; Rousselière and Rousselière, 2017). 57.1% of the respondents thought that the use of cisgenesis to require fewer pesticides in cultivation should be encouraged, and 31.4% approved of the use of transgenesis for this purpose (Hudson et al., 2015). Studies carried out by research groups in Denmark (Edenbrandt et al., 2017; Edenbrandt, 2018) and Italy (De Marchi et al., 2019; De Marchi et al., 2020) also showed that consumers value GM technologies if they lead to a positive effect on the environment. For example, a willingness-to-pay study of samples of 713 (Edenbrandt et al., 2017) respectively 843 (Edenbrandt, 2018) consumers resulted in the following preference order:

(i) organic
(ii) cis- or transgenic with environment benefits (pesticide-free crop cultivation)
(iii) conventional
(iv) cisgenic
(v) transgenic

A similar picture emerged from a more recent Eurobarometer survey, which also includes items on gene editing. When asked about the most pressing risks for food safety in 2019 with a list of fifteen topics, GM ingredients in food or drinks ranked on place 8 (27% of respondents expressed concern), while genome editing (GE) emerged as the one Europeans were least (4%) concerned about (European Commission, 2019a, p. 40). To this group, the most pressing issues regarding food safety were antibiotic, hormone or steroid residues in meat (44%), pesticide residues in food (39%), and environmental pollutants in fish, meat or dairy (37%). Still, how this plays out in consumer acceptance is not fully clear. Some studies assessing the consumers’ willingness-to-pay found no significant differences in the consumer views on GMOs versus GE crops (e.g., Shew et al., 2018). Other studies found small (Delwaide et al., 2015) or considerable differences between the two techniques (Marette et al., 2021).

However, interpreting this as broad change of public opinion towards a more positive assessment of biotechnologies (Woźniak et al., 2021) appears to be premature. Rather, it appears that the European publics are not yet fully convinced of the benefits of genome editing applications (Bundesinstitut für Risikobewertung, 2017; Dallendörfer et al., 2022). What has emerged from opinion polls and survey studies on GM also holds true for genome editing: The crucial factor influencing the attitude towards various biotechnological methods is the type and purpose of modification (Mielby et al., 2012; LIS Consult, 2019). A recent study showed that across the countries covered (Austria, Canada, Germany, Italy, and the US; *n* = 3,698), a hypothetical HIV resistance in humans was considered the most acceptable, followed by mildew resistance in wheat, a virus resistance in pigs (PRRSV), and the production of allergen-free milk. These, in the widest sense, health-oriented applications were considered to be more acceptable than increased muscle growth in cattle (Busch et al., 2021).

Drawing on results of willingness-to-pay and willingness-to-consume (WTC) studies as an indicator of consumer acceptability led study authors to suggest a preference order conventional—cisgenic/genome edited—transgenic (Delwaide et al., 2015).

However, the experiences of the European debate on GMOs in the 1990ies and early 2000 suggest that a renewal of the once powerful alliance between certain Member States and influential groups in civil society and organic farming is not unlikely. In any case, one could expect a protracted debate difficult to resolve in the EU multi-level system.

## Developments in other regions

Another European country, the United Kingdom, has just recently announced amendments to the legislation (UK Parliament, 2022). The Government is proposing GEOs to be exempted from GMO regulations, provided the genetic changes could also occur naturally or via existing conventional breeding techniques. As a first step, this exemption would apply to field trials in England only. In a second step, an amendment of the legal definition of GMOs is planned. This comes at a time when a recent online survey among UK residents showed the acceptability of GE plants was a bit higher than of GM plants (49% resp. 44%) (Ipsos MORI, 2021).

A very recent development likely to drive plant innovation and regulatory developments globally is the issuing of guidelines in China on genome-edited plants (U.S. Department of Agriculture, 2022). Like Australia’s recent decision on SDN1 events, in China it is recommended that genome-edited plants that do not contain exogenous genes, can be considered for safety evaluation. Safety evaluation involves review of the plants details and data related to biosafety and food safety by the Chinese Ministry of Agriculture and Rural Affairs. If the modified trait does not increase environmental or health risks an application can be made for a reduced testing package.

## Discussion and conclusion

The broader picture emerging from the developments reviewed in previous sections indicates new dynamics in social acceptability of GEOs in (up until now) some of the countries described above.

The main drivers for policy change in non-adopters seem to be similar in all jurisdictions explored, although with some differences across the countries studied. Strong pressure emerges from international trade with agricultural commodities and food/feed between countries without authorisation and labelling requirements for GEOs and others, which (still) do require GMO-type pre-market approval, labelling, and traceability. The technical inability to identify/measure certain types of GEOs makes it impossible to enforce the legislation and is expected to be associated with a variety of economic and legal risks (Grohmann et al., 2019). A number of genome-edited plants have already been commercialised, and more are to be expected. Besides United States, Canada, and Australia more than 15 jurisdictions have so far exempted certain GEOs from GMO legislation or established fast-track procedures, among them important agricultural exporters, e.g. Brazil and Argentina (Menz et al., 2020; Entine et al., 2021; Turnbull et al., 2021). As more jurisdictions will join this group, pressure will increase on jurisdictions, which regulate GEOs the same way as GMOs. Thus, the support of established public policies amongst politicians and certain stakeholders has declined, marking a major change in the socio-political dimension.

There is also increasing awareness that innovation in agriculture needs to address problems such as climate change which urgently require policy action. These new technologies are becoming more accessible for small to medium sized plant breeding business, as well as used for smaller seed markets, and therefore are likely to lead to a more diverse range of breeding innovations (Whelan et al., 2020; Purnhagen and Wesseler, 2021). Plant genome editing has potential to make important contributions to more sustainable agriculture, by developing plants that have clear environmental benefits (drought resistance, increased shelf-life etc.) and by contributing to biodiversity. In light of these developments, there is also political pressure mounting to utilize the potential of genome editing (Anders et al., 2021), another reason behind the declining support of existing regulations in non-adopting countries (and beyond).

Although some authors diagnosed a change in citizen/consumer views on GMO-related topics (An et al., 2019; LIS Consult, 2019; Muringai et al., 2020; Ipsos MORI, 2021; Ortega et al., 2022), the empirical studies reviewed in this paper (overview and references provided in Table 4) do not support this diagnosis—neither for the adopters nor for the non-adopters. When asked to state preferences, respondents are generally still opposed to GMOs and also opposed to GEOs—although to a lesser extent. Yet, this has to be taken with a grain of salt. Experiments observing actual purchasing behavior showed that stated preferences (SP) of consumers with regard to GMO food products differ from their revealed preferences (RP): people are more likely to purchase GMO food products than they are to say they would (Mather et al., 2012; Sleenhoff and Osseweijer, 2013). This is in line with findings from behavioral psychology that suggest that people tend to overstate their preferences, especially when it concerns products with moral implications (Johansson-Stenman and Svedsäter, 2012).

**TABLE 4:** Recent studies on attitudes of citizens, consumers, or stakeholders towards genome-edited plants considered in this review.^a^

Geographical scope	Method	Target group	References	Reviewed in Beghin and Gustafson (2021)
Australia	Questionnaire	Citizens	Batalha et al. (2021)	N
Canada	Choice experiment	Consumers	Muringai et al. (2020)	Y
Canada	Questionnaire	Consumers	Yang and Hobbs (2020)	Y
Canada	Questionnaire	Value chain stakeholders	Smyth et al. (2020)	N
Canada	Survey	Consumers	Vasquez Arreaga (2020)	Y
Canada	Survey, choice experiment	Consumers	An et al. (2019)	Y
Canada, United States, Austria, Germany, Italy	Questionnaire	Citizens	Busch et al. (2021)	Y
China	Choice experiment	Consumers	Ortega et al. (2022)	Y
Europe, United States, Japan	Survey	Value chain stakeholders	Jorasch (2020)	N
Finland	Interviews, survey	Citizens	Wessberg et al. (2021)	N
France	Qualitative sorting exercise	Citizens, value chain stakeholders	Debucquet et al. (2020)	N
France, United States	Choice experiment	Consumers	Marette et al. (2021)	Y
Germany	Discourse analysis	n.a.	Siebert et al. (2021)	N
Germany	Focus group interviews	Citizens	Bundesinstitut für Risikobewertung (2017)	N
Germany	Macro-economic simulation	n.a.	Maaß et al. (2019)	N
Germany	Qualitative interviews	Citizens	Friedrich (2020)	N
Germany	Questionnaire	Citizens	Dallendörfer et al. (2022)	N
Japan	Discourse analysis, participant observation	n.a.	Yamaguchi (2020)	N
Japan	Questionnaire	Citizens	Farid et al. (2020)	Y
Japan	Questionnaire	Citizens	Hibino et al. (2019)	N
Japan	Questionnaire	Consumers	Kato-Nitta et al. (2021)	Y
Japan	Questionnaire	Citizens, value chain stakeholders	Kato-Nitta et al. (2019)	Y
Japan	Questionnaire	Consumers	Otsuka (2021)	N
Japan	Twitter analysis	Citizens	Tabei et al. (2020)	Y
Netherlands	Questionnaire and interviews	Citizens	LIS Consult (2019)	N
Netherlands, Belgium	Questionnaire	Consumers	Pellens (2019)	N
New Zealand	Qualitative interviews	Citizens	Hudson et al. (2019)	N
Norway	Questionnaire, focus groups	Citizens	GENEinnovate (2020)	N
Switzerland (German-speaking area)	Choice experiment, online, consumer panel	Consumers	Saleh et al. (2021)	Y
United Kingdom	Twitter analysis, workshops	Citizens	Smith and Samuel (2018)	N
United Kingdom	Workshops, online survey	Citizens	Ipsos MORI (2021)	N
United States	Facebook analysis	Citizens	Walker and Malson (2020)	N
United States	Questionnaire	Citizens, value chain stakeholders	Calabrese et al. (2021)	N
United States	Questionnaire	Consumers	Caputo et al. (2020)	Y
United States	Twitter analysis, metaphor analysis, questionnaire	Citizens	Hill (2020)	N
United States, Canada, Belgium, France, Australia	Choice experiment	Consumers	Shew et al. (2018)	Y

a This table is the result of a multi-stage literature screening process. In a first step, we searched established literature databases (Scopus, Web of Science, Google Scholar) with a large selection of keywords related to gene or genome-edited plants, cisgenesis, New Plant Breeding Techniques, and New Genomic Techniques. After a first screening on whether the papers included an empirical study of attitudes or opinions, we followed the snowball strategy and included selected references cited in the papers. From this still growing database, this table only shows those studies concerned with GEOs. Further, articles not reporting new data (e.g., reviews) are not included. n.a., non applicable; Y, yes; N, no.

At any rate, public opinion or consumer demand do not appear to be relevant drivers for policy development at present. Summarizing the recent dynamics in socio-political acceptability of genome-edited plants, there has been an increasing awareness that the legal regulations in non-adopting countries are no longer fit for purpose, leading to a decrease in support of current public policies amongst some politicians and stakeholders. However, the public opinion on the biotechnologies has hardly changed; a significant change, however, has occurred in public awareness regarding the importance of measures to prevent a climate catastrophe.

In the field of plant biotechnology, the introduction of genome editing techniques had direct effects first and foremost on the positions of stakeholders and political decision-makers, and less on the opinions of citizens. Studies that compare citizens’ or consumers’ perceptions of GMOs, GEOs, and other breeding techniques exist, but their findings have to be taken with caution. Across the countries covered, most people do not know about similarities and differences between GMOs and GEOs. Therefore, studies interested in consumers’ or citizens’ views on these techniques have to provide respondents with definitions. The task to write up definitions that are, at the same time, scientifically correct, straightforward and understandable, and ideologically unbiased is a huge challenge for researchers.

One aspect, however, that emerges very clearly from the comparison of consumer studies is that acceptability is significantly higher if the edit leads to sustainability benefits. This has markedly changed over the last years and can be interpreted as reflecting the increasing awareness of the challenges posed by the climate crisis and the increasing sense of urgency for action. This can be taken as an indication that—in parallel to the changes discussed in terms of socio-political acceptability—there is also a change in potential market acceptability. While it is too early for drawing firm conclusions, it seems possible that consumers in non-adopting countries might be open to purchase food produced from genome-edited plants if it has a clear environmental benefit.

Against this backdrop, policies have already been changed in one country of the non-adopter group. Japan has exempted GEOs without recombinant DNA (SDN1) from GMO legislation. Here, the change was driven by policy-makers without significant involvement of food value chain actors. Japanese retailers do not yet seem ready to accept the first genome-edited products which have entered the market. These products are made by start-ups and marketed via internet directly to consumers—thereby bypassing traditional gatekeepers in the food-value chain. In terms of the theoretical frame used for this review, this can be interpreted as yet another indication of an increased acceptability of consumers of food products derived from genome-edited plants (market acceptability).

Initiatives with similar goals can be observed in the EU, Norway, Switzerland, and the UK. Interestingly, in some countries, e.g., in Norway and Switzerland, companies and business associations active in the food/feed chain have not waited for policy makers to set the stage. In these countries, activities are led by stakeholders who previously strove for GM-free food/feed. Value chain actors made use of an organized protected forum to explore views and to coordinate steps towards more openness to GEOs, in Norway the review of corporate GMO policies, in Switzerland the activities in the context of the “Varieties for Tomorrow”. Seen against the backdrop of the triangle of social acceptability, we can analyze that market actors of the food value chain became proactive, but targeted socio-political acceptability rather than their own market sphere. This can be interpreted as a signal to policy makers and other stakeholders—intending to demonstrate their support of efforts towards increasing the socio-political acceptability of the technology and the related policies. Moreover, it might also imply that once socio-political acceptability is sufficiently stable, food value chain actors are prepared to take steps towards also stabilizing market acceptability, within their primary sphere of activities, the food value chain.

To be clear, these opening-up initiatives are confronted with challenges, which seem to differ between jurisdictions and appear to be particularly problematic for the European region. Some countries in this region have long pursued a no-GMO policy and strongly advocate a GEO = GMO policy. GMO-critical civil society groups partly in coalition with organic farmers, small-holder associations, and green parties were and still are active and influential on policy development in many European countries (e.g., Bernauer and Meins, 2003; van Schendelen, 2003; Kuntz, 2014; Tiberghien, 2015, Tosun and Schaub, 2017; Tosun and Varone 2021). Considering the requirement of the EU treaties to achieve majorities for amending legislation and the still diverse and polarized views, any policy change is likely to require compromises. Compromises in the European context could possibly include sustainability requirements and/or labelling needs—as indicated in European Commission documents (European Commission, 2021a; European Commission, 2021b). Here, considerable challenges and pitfalls are likely when implementing such compromises. E.g., how can sustainability requirements be linked to market access? Drawing on the experience with the disagreements in the GMO risk assessment when discussing broader socio-economic impacts in the EU (reviewed in Spök, 2010) a mandatory sustainability assessment similar to the Norwegian one is highly unlikely to work smoothly in the EU context.

Another challenge is ironically associated with established policies to make agriculture more sustainable. For the EU context, for instance, this includes a goal to reach a 25% share of total arable land dedicated to organic production by 2030 (European Commission, 2019b; European Commission, 2020a) compared to an average share of 8% in 2019. Even this 8% share is associated with total retail shares in the EU of 41 billion Euro (Willer et al., 2022) indicating successful pro-organic policies in the past and consumer demands for these products.

EU legislation does not allow GM ingredients in organic food products but tolerates traces of up to 0.9% authorized GMOs (zero tolerance for unauthorized GMOs). Assuming that GEOs equal GMOs it would still be unclear how to enforce 0.9%. The challenges for organic producers are, however, increasing dramatically, if certain GEOs would be exempted from the legal GMO definition (Purnhagen et al., 2021). In such a scenario, existing EU legislation would no longer require organic producers to avoid ingredients from GEOs. Yet, the world association of organic producers IFOAM, already excluded genome editing, along with other techniques, from organic farming for ethical reasons (IFOAM, 2017). In such a scenario, organic producers are likely to find themselves in a risky business environment. In order to avoid GEO ingredients in their products existing certificate schemes would need to be extended without the possibility for double-checks by accredited laboratories. This is likely to results in additional burden on organic producers, leading to liability issues and affecting consumer trust. As regards European countries, this would affect in particular those which have already reached high shares of organic farming, e.g., Austria, Sweden but also Switzerland.

These challenges, in principle, also apply to GMO-free food products, which, at least in the EU, is a significant market. According to self-estimates, their share in German and Austrian retail amounts to some 10 billion Euro (European Non-GMO Industry Association, 2022). GMO-free production is in most cases guided by private standards and definitions. Therefore, these producers would in principle be more flexible than organic producers to accept food and food ingredients from GE plants if they are not considered GMO. In a scenario where GEOs would still fall under the legal definition of GMOs they would also be flexible to adjust their criteria. It cannot be excluded that labelling schemes would respond differently and thereby compete each other with different versions of GMO-freeness. Therefore, both scenarios would bring considerable challenges for the GMO-free sector.

Even if the remaining non-adopters would open up for GEOs, another challenge remains. As outlined in Table 5 slightly different criteria are emerging in different jurisdictions for exempting GEOs from GMO legislation or regulating them differently. If commodities and food products which do not need a pre-market approval and labelling could only be traded with some but not with other countries, this is likely to hamper international trade. The enforcement of legislations would become highly complex if, for instance, some countries were exempting SDN1 while other are also exempting SDN2; if some need proof of absence of off-targets while others do not. Efforts to internationally harmonise definitions and rules, therefore, would be the only way to avoid such a situation.

**TABLE 5:** Scope of legal exemptions or amendment for genome-edited plants established or proposed in the jurisdictions considered.

Jurisdiction/status in the legal process	Scope of legal exemption/amendment	Permission/notification needed [P, N, none]	Risk assessment requirements [GMO-RA, specific RA, none]^b^	Labelling requirements [GMO labelling, specific, none]^c^
United States/established	Cisgenesis, intragenesis Deletion(s) of any size; Targeted substitutions of a single base pair; edits from sequences which are known to correspond in the plants natural gene pool. GMO with known plant/trait interaction	P	None	None
Canada/established	Cisgenesis (not novel)	N	GMO-RA^a^	None
Australia/established	No DNA inserted (SDN1); RNAi (not inserted in genome)	P	None	None
Japan/established	No DNA/RNA inserted, e.g. SDN1; cisgenesis	N	None	None
New Zealand/established	No exemptions	P	GMO-RA	Not yet specified
EU/discussion proposal	Cisgenesis, SDN1, SDN2	Not yet specified	Not yet specified	Not yet specified
Norway/discussion proposal	Cisgenesis, intragenesis, SDN1	N^d^	None	Specific
Switzerland/discussion proposal	Absence of transgenes	Not yet specified	Not yet specified	Not yet specified

a If considered novel.

b GMO-RA: same risk assessment as for GMOs; specific: specific risk assessment required - would be helpful to add some details on the specific procedure in the footnote to the table.

c GMO labelling: same labelling required as for GMOs; specific: specific labelling required—please, describe in the footnote to the table.

d Proof of absence of off-target mutations required.

GMO-RA, same risk assessment requirements as for GMOs; GMO-labelling, same labelling requirements as for GMOs; P, permission; N, notification.

Looking again at the broader picture and reflecting on the prognoses of Ishii and Araki (2017) it appears that they correctly anticipated what happened at the policy level in Japan and New Zealand. They also seem to have correctly predicted the path the United Kingdom has taken post-Brexit. As regards the EU the situation is however, still unclear: considering the recent development described in this paper and the opening-up initiatives in Norway and Switzerland as indicators, it still seems possible that the EU could amend its GMO legislation to establish a more enabling legal environment for certain genome-edited plants and derived products. Considering the large EU trade volume for agricultural commodities—185 billion Euros exports and 143 billion Euros imports (Eurostat, 2021)—this is likely to have an impact on the policy development in countries with a significant share in food trade with Europe.

A powerful narrative is emerging, that focuses on how genome-edited crops can be a tool to reduce the impact of our agriculture on the climate and environment. Food value-chain actors, which have been extremely shy in public arenas and navigated on the markets in an ultra-precautionary way, are becoming proactive towards opening-up for GEOs on the level of public policy. This may serve as a wake-up call for certain environmental groups and organic farmers to review and reconsider their policies on genome editing. Considering the urgency of the happening climate crisis, we cannot afford to continue with the carte-blanche pro-con discussions in the same way as in the last 30 years.

## Footnotes

1 A different interpretation by van der Meer et al. (2021) is that the ruling has to be interpreted in a more narrow way. Following their line of thoughts, it is unclear whether at all and how genome-edited organisms are covered by Directive 2001/18/EC.
